# Policy options for deploying anti-malarial drugs in endemic countries: a population genetics approach

**DOI:** 10.1186/1475-2875-11-422

**Published:** 2012-12-17

**Authors:** Tiago Antao, Ian Hastings

**Affiliations:** 1 Liverpool School of Tropical Medicine, Pembroke Place, Liverpool, L3 5QA, UK; 2 Current address: Wellcome Trust Centre for Human Genetics, University of Oxford, Oxford, OX37BN, UK

**Keywords:** Malaria, Plasmodium falciparum, Drugs, Resistance, Treatment, Policy, Adherence

## Abstract

**Background:**

Anti-malarial drugs are constantly exposed to the threat of evolving drug resistance so good stewardship of existing therapy is an essential component of public health policy. However, the widespread availability of numerous different drugs through informal providers could undermine official drug deployment policies. A policy of multiple first-line therapy (MFT) is compared with the conventional policy of sequential drug deployment, i.e., where one drug is used until resistance evolves and then replaced by the next drug in the sequence.

**Methods:**

Population genetic models of drug resistance are used to make the comparison; this methodology explicitly tracks the genetics of drug resistance (including, importantly, recombination in the sexual stage, intrahost dynamics, and direction of linkage disequilibrium).

**Results:**

A policy of MFT outlasts sequential application providing drug usages are low to moderate, and appears not to drive widespread multi-drug resistance. Inadequate dosing is an even more potent driver of drug resistance than the MFT/sequential policy decision.

**Conclusions:**

The provision of MFT as a deliberate policy can be encouraged provided overall treatment rates are low or moderate (less than around half of malaria infections are treated) and the *ad hoc* provision of MFT through the private sector may be tolerated. This must be fully supported by education to ensure people take adequate doses of each of the drugs.

## Background

Malaria imposes considerable strains on local health infrastructures, where well over half of paediatric hospital admission may be attributable to malaria (e.g., [1]), places significant financial burden on families and individuals who are not hospitalized [2], and is estimated to slow national economic growth by around 1.7% per annum. The provision of effective anti-malarial drugs is therefore a mainstay of public health policies aimed at reducing morbidity and mortality [3]. Most countries now adhere to the mandatory deployment of anti-malarial drug combinations, invariably in the form of artemisinin combination therapy (ACT), as a means of delaying the evolution of drug resistance [4,5].

There are currently several effective forms of ACT based around amodiaquine, lumefantrine, mefloquine, piperaquine, sulphadoxine-pyrimethamine (where the latter is still effective) and possibly CQ [6]. Current drug deployment policies, hereafter termed “sequential”, make use of a single first-line therapy which is used until the level of treatment failure rises above an acceptable level (currently 10% [7]), at which time the drug should be replaced with a new treatment. An alternative strategy would be to deliberately deploy multiple first-line therapy (MFT) simultaneously, resulting in malaria infections being treated with different drugs. Widespread provision of different drugs through the informal, private sector may already result in a *de facto* situation of MFT in many countries [8].

An innovative evolutionary-epidemiological study [9] suggested that MFT would result in longer overall periods of drug effectiveness. This was based on the ecological argument that it is more difficult for organisms to evolve in a heterogeneous environment caused, in this case, by different drug treatments, than in a homogenous treatment environment associated with single drug use. This ecological argument is sound but the study was unable to incorporate three important facets of malaria epidemiology. Firstly, the threat posed by the emergence of multi-drug-resistant genotypes. Boni *et al.*[9] were prevented, by technical reasons, from an explicit investigation because their methodology assumed random breeding among malaria parasites within the whole parasite population. This greatly overestimates the extent of sexual recombination in the malaria population because, in reality, recombination can only occur between malaria clones co-infecting the same human. The small number of clones in a person (usually one to five) means self-fertilization is frequent (typically >50% [10,11]) and, furthermore, only resistant parasites are present in a person following drug treatment so sexual breakdown of resistant haplotypes is much lower. Sexual recombination determines the extent of linkage disequilibrium (LD), which has long been known as a key driver of multi-drug resistance [12,13]. LD is such a critical parameter because if resistance mutations become statistically associated in the same parasites then each resistance mutation essentially ‘hitchhikes’ with the others because multi-drug-resistant haplotypes can survive treatment with any drug, enabling multi-drug resistance to rapidly sweep through a population. The second facet was to ignore the effects of intrahost dynamics: if a treated individual contains several malaria clones then drug-mediated removal of sensitive clones may boost the transmission of any surviving resistant clones by removing competition. This is one of the three key drivers of anti-malarial drug resistance [5,14] also known as ‘competitive release’ [15]. The third facet was the assumption that an infection met only one drug during its infective lifespan. The reality in many clinical settings is that drugs are used presumptively to treat any undiagnosed fevers and drugs are widely available outside the formal health service; most non-artemisinin anti-malarial drugs have long half-lives and persist for weeks after treatment, consequently many patients harbour residual drug from previous treatments [16,17]. If the residual and treatment drugs are different (as will frequently occur in areas utilizing MFT) then the parasites must be resistant to both drugs: resistance to the residual drug allows the infection to become established, while resistance to the second allows it to survive direct therapy. The policy of MFT therefore slowly morphs into a *de facto* policy of combination therapy (CT) that is known to be a potent driver of multi-drug resistance. In summary, a worst-case scenario under MFT is that different resistance mutations become genetically associated so that multi-drug-resistant parasites rapidly arise that are fully resistant to all drugs in the MFT arsenal. It is impossible to recommend the use of MFT without quantifying this risk especially given the high-profile consequences of multi-drug resistance in other infections such as tuberculosis and Methicillin-resistant *Staphylococcus aureus* (MRSA) [18,19].

It is interesting to note that a similar debate is taking place in bacteriology, where the relative merits of antibiotic rotations and mixing are being investigated (see, for example, [20-22]). Several of the principles are the same as for malaria treatment but there are important biological differences that preclude a direct comparison. Bacteria acquire resistance extremely quickly, and the main concern in therapy is preventing the *de novo* emergence of resistance from within a bacterial infection; in contrast, with the exception of atovaquone, *de novo* resistance to most anti-malarial drugs occurs extremely infrequently. Bacteria also have very different genetics: a relatively high mutation rate, meiosis is absent so that genetic recombination is less formalized, and horizontal gene acquisition may occur.

Herein, an explicit population genetic model is used to compare the policies of sequential drug use and MFT in the treatment of malaria and address the key issue of most contemporary concern to policy makers: the extent to which they should encourage, tolerate, or even attempt to suppress the parallel deployments of MFTs (in the same way that they suppress the deployment of artemisinins as monotherapy [4]).

The primary purpose of this study is to consider the risk posed by MFT in driving LD and multi-drug resistance. However, the methodology was extended to address several other operational questions omitted from the previous MFT study. Do different clinical/epidemiological settings, which can alter the genetic basis of resistance, affect the relative advantages of sequential and MFT applications? Is MFT always the best strategy in all epidemiological settings? If MFT is to be tried, should it first be trialled in high or low transmission areas? How does the presence of intrahost dynamics and competitive release [14,15,23] affect the results? Would a strategy of full combination therapy, i.e., combining all available drugs in a single therapy, be an even better strategy?; it is unlikely that any policy maker would be brave enough to consider this option, but it provides a useful theoretical baseline (see later).

## Methods

OgaraK [24] was used to carry out the comparisons. This is a population genetics simulator of the emergence and spread of drug resistant malaria that incorporates multiple genetic loci, sexual recombination, LD, differing levels of multiplicity of infection (MOI, see below) and different genetic models of how resistance is encoded.

Population genetic models for malaria have to address several non-standard features of its biology; these are briefly re-iterated here together with the conventional assumptions made during their modelling. Malaria parasites are haploid and reproduce asexually in humans. Humans are repeatedly bitten by infected mosquitoes, which leads to several genetically distinct malaria clones being present in the blood at any given time. The number of simultaneous infections in a human is termed the multiplicity of infection (MOI), which typically ranges from one to 12. Malaria parasites are briefly sexual and diploid in the mosquito vector, where they reproduce sexually in the conventional eukaryote manner, i.e., through crossing-over and recombination between chromosomes. Crucially, the sexual phase can only involve parasites ingested through the same blood meal (mosquitoes feed approximately every three days so mating between parasites in blood meals obtained in separate bites is assumed to be impossible). It is assumed that each blood meal is taken from a single human so the number of mating options inside the mosquito is dependent on the MOI of the human providing the blood meal (interrupted feeding followed by the mosquito resuming feeding on a different human does occasionally occur but this essentially just increases the MOI in the meal). This creates an environment which departs from standard population genetic models, namely a highly substructured population where mating can only occur between the small number of different parasite clones within a blood meal. Sexual union and recombination between parasites originating from different clones, ‘outcrossing’, results in re-arrangement of genetic material and production of progeny with novel genotypes but recombination between parasites originating from the same clone, ‘selfing’, results in genetic shuffling of identical haploid genotypes so the progeny are genetically identical to the parental clone [25].

Infections are assumed to have equal infectivity and mate at random inside the mosquito so that if there are *n* clones in a human then selfing rate is 1*/n* and outcrossing rate is 1-1/*n* and competitive release [13,23] is assumed to occur. It was assumed, in common with most malaria genetic modelling, that parasite clones were unrelated within humans although recent evidence suggest this may not always be the case [26]; in this case the recombination rate between loci, *r*, needs to be scaled by the degree of unrelatedness, i.e., *r*(1-*F*) where F is the relatedness of the clones. A crucial difference between areas of high and low malaria transmission is the MOI: high malaria inoculation rates in areas of high transmission intensity leads to higher MOI, so mosquitoes frequently ingest unrelated parasites resulting in increased levels of sexual recombination [27]. MOI is, therefore, a reflection of transmission intensity.

Different infections may have different drug-resistance profiles. The genotypes of each infection were simulated assuming that all loci are physically unlinked, a realistic assumption for most loci known to encode malaria drug resistance [28]. A simplifying assumption is also made that that there is no cross-resistance among drugs (i.e., a single mutation cannot encode resistance to more than one drug in the therapeutic arsenal), but note that this is not applicable to all known cases [29]. Each locus can have two alleles: resistant and sensitive. Resistant alleles will incur a fitness penalty if they are not required for survival [i.e., in untreated hosts, in hosts treated with a drug for which the mutation cannot encode resistance or in cases where the epistasis mode (see below) does not require all mutations]. Parasites in infected humans may remain untreated (an environment where sensitive infections are fitter), treated in humans who have little or no host immunity (where a weaker epistasis mode suffices to confer resistance), or treated in humans who are semi-immune to infection (requiring the strong mode of resistance). Parasite multi-locus genotypes can vary from sensitive to all drugs to resistant to all drug treatments available and having all mutations (multi-resistant).

For all simulation scenarios the fitness penalty associated with each resistant mutation and the amount of drug treatment (defined as the percentage of infected humans that were treated) were both varied between 0 and 100% in increments of 2%. Genotypes with multiple mutations incur a multiplicative fitness penalty. Four different MOI scenarios were simulated. Two simple scenarios where each human had MOI fixed at two (low transmission) and four (high transmission). Two more realistic scenarios were also investigated: one (low transmission) where 50% of human hosts had a single infection (MOI=1) and the other half had MOI=2, and another (high transmission) scenario where humans had MOI described by a Poisson distribution with a conditional mean of 2.3 truncated at a maximum MOI of seven [23]. The simpler MOI scenarios qualitatively capture the results of the more complex ones (results not shown), so only the results from the simple MOI distributions are presented. All these results can easily be replicated as ogaraK is made publicly available [24] and can simulate all the scenarios above.

It is assumed that resistance to all therapies exist at very low frequencies at the onset of the simulation. The emergence of resistance and its implications on therapy effectiveness have been studied elsewhere [9,30]. This approach is complementary as it attempts to understand the spread, rather than the origin, of existing resistance. Furthermore resistance to most drugs is now widespread, and has probably emerged also for artemisinins [31] and in many cases *de novo* resistance has arrived in human population via migration [32], not local mutation.

These analyses of sequential drug deployment did not allow the option of re-using a drug after all possible therapies have been exhausted. Fitness costs associated with resistance may decrease the frequency of the resistance mutation once the drug has been withdrawn to the extent that a once-failing drug recovers its efficacy and could, in principle, be re-inserted into the sequence. This option was not considered because this possibility is highly dependent on the fitness penalty associated with the resistance mutation and the length of time since it has been withdrawn. Fitness effects seem to vary considerably, the *crt* locus involved in CQ resistance seems to incur a higher fitness penalty than the *dhfr* mutations associated with sulphadoxine-pyrimethamine (SP) resistance [33]. The time between replacements in a sequence may allow the mutation frequency to fall, but not to its original basal level, so resistance will probably rapidly spread again from a relatively high basal frequency [6] and it is not operationally feasible for policy makers to switch drugs over a likely timescale of months (hence the realization that even if CQ is redeployed in Malawi, it would be as part of an ACT).

The same caveats apply here as to any modelling study, primarily that it can only investigate a finite, although plausible, set of conditions. Policy makers may wish to develop and refine similar analyses (using ogaraK) specifically calibrated for their own circumstances. A comprehensive search of parameter space was conducted to verify that the conclusions are robust. For instance, most analyses and discussion was made assuming a range of fitness penalties per loci. This was carefully scrutinized through comparisons with simulations with no fitness penalty. Other assumptions do require future study: (i) if several different ACTs are used then there is likely to be a partially shared resistance basis (on the artemisinin derivative) and further work is needed in modelling drugs with partially shared resistance; (ii) some drugs, notably CQ and amodiaquine [34], do share the same resistance loci but the mechanism of resistance seems to be different – even opposite and (iii) residual drug levels play a critical role in the emergence and spread of resistance [35] but have not been formally included except as a factor resulting in *de facto* CT. It is important that policy makers understand the limitations and assumptions of this (and any other) models of resistance.

The possibility of cross-resistance between drugs or its converse, resistance “antagonism” (e.g., the *pfcrt* K76T mutation which encodes increased resistance to CQ but increased sensitivity to lumefantrine [36]), is an interesting question. This was not considered explicitly here for three key reasons. Firstly, it is likely that people designing a policy of MFT would avoid using drugs to which there was extensive cross-resistance as this, intuitively, would negate its main advantage in slowing the spread of resistance. Secondly, the effect would have to be quantified and there is no guarantee that correlations in resistance caused by single genes (e.g., [37]) would be the same as that produced by standing genetic variation prior to resistance spreading (e.g. [38]). Thirdly, most, if not all, models of multi-drug resistant malaria ignore the possibility of cross resistance so the results presented here are directly comparable to previous work.

An important feature of ogaraK is that when more than one locus encodes resistance to a single treatment, it is able to incorporate different epistasis modes among the resistance loci [13]. It is assumed that two resistance loci determine resistance to each drug and that two drugs are deployed so that a resistance genotype consists of four independent loci. The epistasis modes are as follows.

*‘Full epistasis’:* parasites must carry resistant alleles at both loci to survive treatment by a drug.

*‘Duplicate gene function (DGF)’*: parasites carrying a resistance mutation at either locus can survive treatment by a drug.

*‘Mixed mode’:* Half of the treated patients have no host immunity, thus infections will be able to survive treatment if the most important locus has a resistant allele (asymmetrical epistasis), the other half will be immune, therefore only parasites that have both resistant alleles will survive treatment (full epistasis). Asymmetrical epistasis reflects the resistance mechanism observed in both SP and CQ where the ‘most important’ loci are *crt* and *dhfr* respectively with *mdr1* and *dhps* playing a lesser role in encoding resistance.

Sequential application, MFT and combination were investigated using these different modes of epistasis to capture the clinical/epidemiological variation known to be important in field conditions. For example, a person who takes a full drug dosage and/or is semi-immune is a very harsh environment for parasites and they may require mutations at all loci to survive (described by ‘Full epistasis’). Conversely, a patient who takes a sub-optimal dose and/or is non-immune to malaria constitutes a relatively benign treatment environment and parasites may survive if they posses only a resistant mutation at a single locus (described by DGF or Asymmetrical Epistasis). Full epistasis is deemed a “strong” mode as it requires all mutations for resistance; conversely DGF and Asymmetrical Epistasis are called “weak”.

The spread of resistance was tracked over 200 parasite generations (where a generation encompasses one complete malaria life cycle mosquito→human→mosquito); there are likely to be around five generations per year [39,40] so the simulations last approximately 40 years which easily encompasses the timescales likely to be considered in long-term planning. In order to compare policy duration it was assumed that a sequential policy lasts until the last drug is removed from circulation. A drug is replaced as soon as an average of 10% of infections are resistant to it. For MFT and combination therapy it was assumed that a policy stops being effective as soon as 10% of all infections resist treatment. This figure of 10% was chosen because the World Health Organization (WHO) recommends a change of treatment regimen when cure rate falls below 90% [7].

## Results

**Figure 1:**
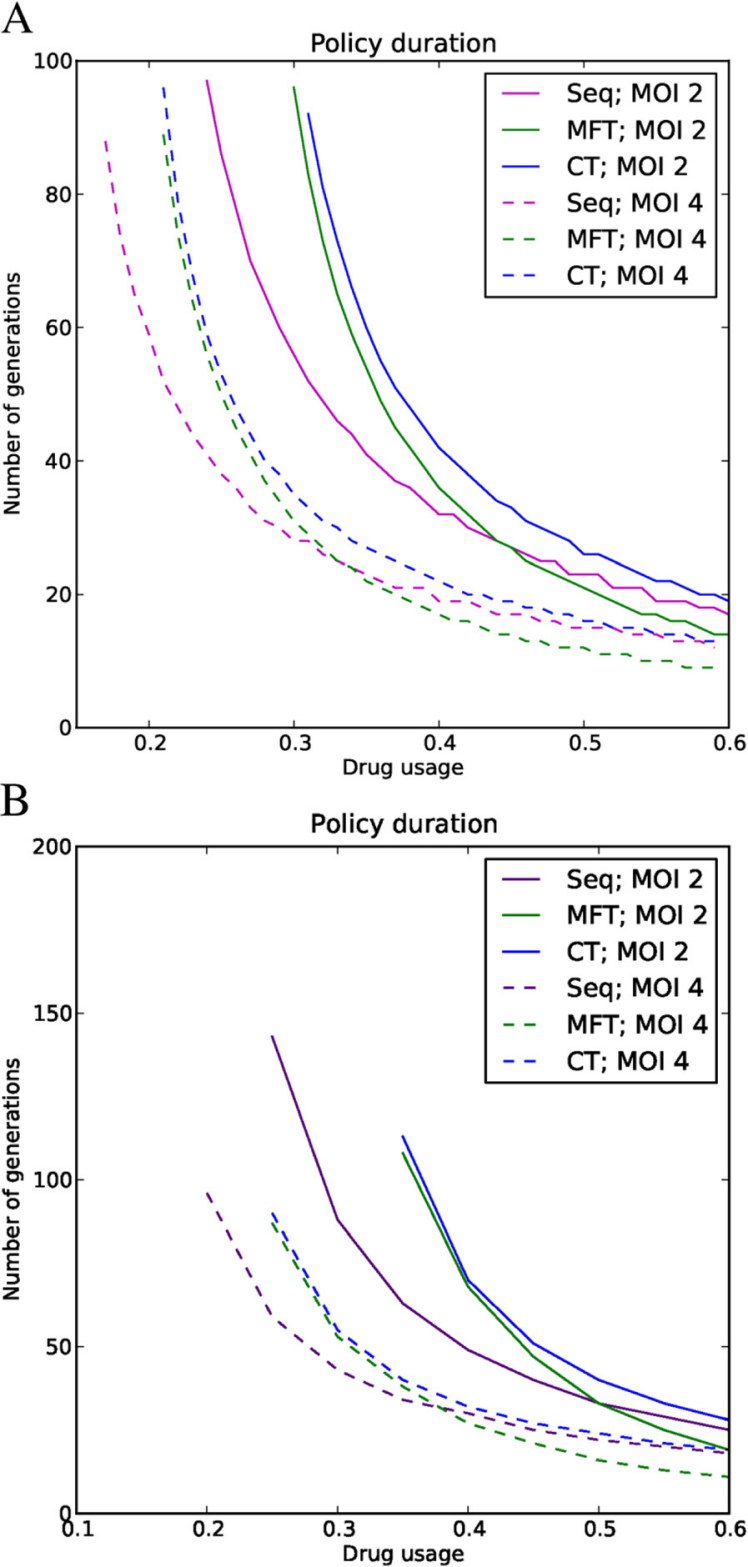
**Impact of policy and multiplicity of infection (MOI) on useful therapeutic life; the latter is plotted on the Y axis and is defined as the number of parasite generations that elapse before overall drug failure rates reach 10%.** It is plotted as a function of drug usage (the proportion of infections that are treated) on the X-axis. The chart compares combination therapy (CT), MFT and sequential (Seq) deployment policies with MOIs of 2 and 4 and with a fitness penalty of 10%. MFT and CT perform better than sequential policies at lower drug usage but MFT marginally worse with high drug usage. All three deployment policies last longer with lower MOIs. (**A**) Two drugs are available. (**B**) Three drugs are available; the lines start abruptly because for lower drug usage the policies last longer than the 200 generations simulated.

**Figure 2:**
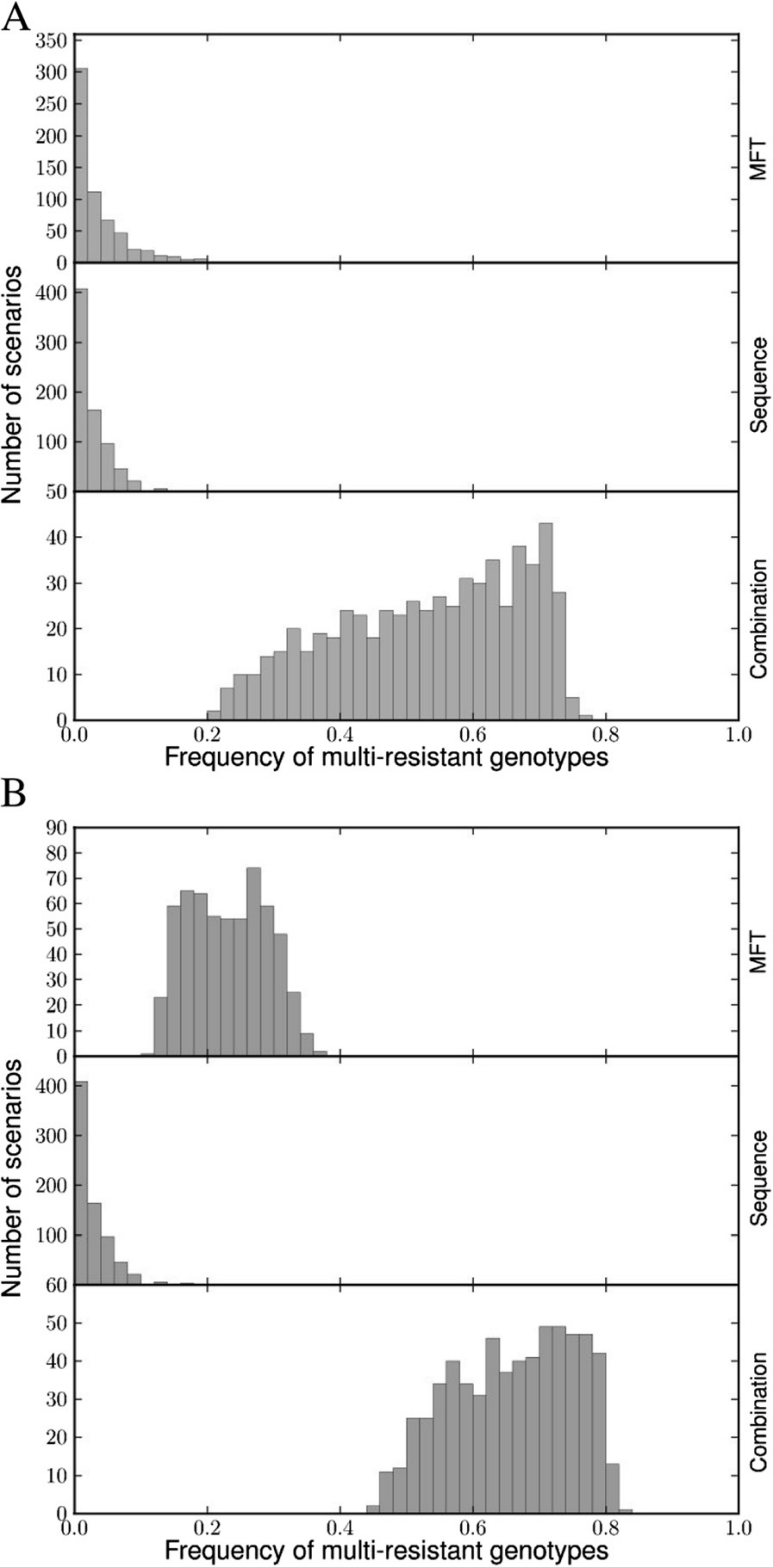
**The impact of deployment policy on the spread of multi-drug resistant malaria: the distribution of multi-drug resistant genotypes at the policies’ end-of-life.** MFT and sequential application show similar patterns where most genotypes are not multi-resistant. Combination therapy is qualitatively different as most genotypes at policy end-of-life are multi-resistant. (**A**) End of life is assumed to occur at 10% resistance. (**B**) End of life is assumed to occur at 50% resistance.

MFT policies last longer than equivalent sequential policies for low to medium drug usage (Figure 1). Sequential application performs better when treatment rates are high, but in this case the difference between policies is minor. Varying fitness penalty (including no fitness penalty) has no qualitative effect on these conclusions. Combination therapy resulted in therapeutic lifespan very similar to MFT (Figure 1). The fundamental qualitative difference between combination therapy and the other policies is that CT is a strong driver of multiple drug resistance with frequency of the multi-resistant genotype usually above 50% at the end of its useful therapeutic lifespan (Figure 2A). The multi-resistant genotype is the only one that can resist treatment under combination therapy so most resistant genotypes are multi-drug resistant under this deployment policy. Conversely, the multi-resistant genotype is never the most fit in any human hosts under a policy of sequential application or MFT because this genotype always pays fitness costs for carrying resistance mutations to drugs that are not used to treated the host; consequently multi-drug resistance only becomes frequent, through random genetic association, when resistance to all drugs is high. This random process is qualitatively different with low and high resistance frequencies [13], so the same comparison was made for a much higher threshold of resistance (50%) before a drug is removed. Simulations show that MFT multi-resistant pattern then shifts to an intermediate between sequential application and combination therapy (Figure 2B).

**Figure 3:**
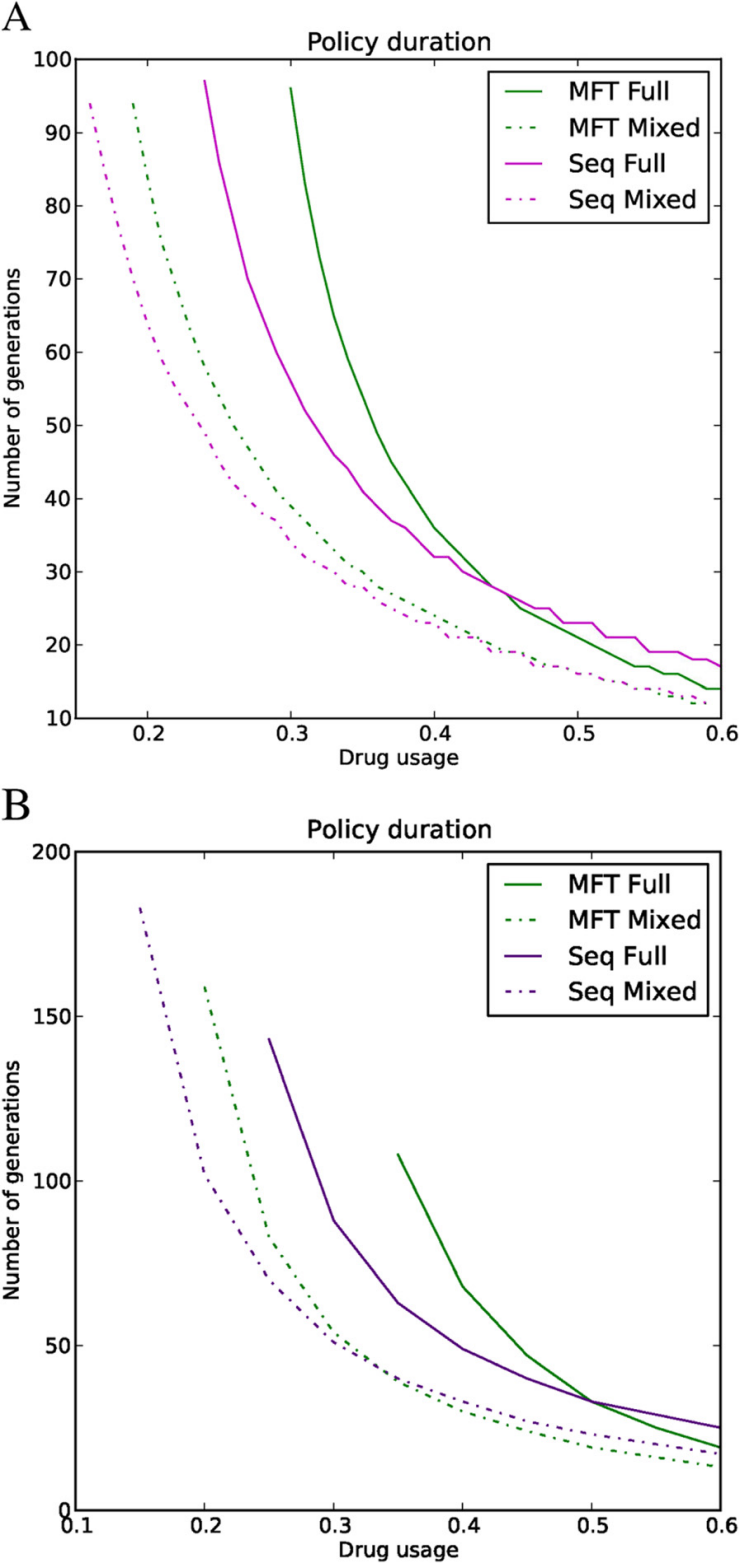
**Impact of policy and epistasis on useful therapeutic life.** Policy duration is plotted on the Y-axis and the drug usage on the X-axis. The chart compares full epistasis with the ‘mixed’ epistatic mode of resistance for MFT and sequential drug application assuming a fitness penalty of 10%. The useful therapeutic life always lasts longer in clinical/epidemiological settings requiring full epistasis rather than mixed model. (**A**) Two drugs are available. (**B**) Three drugs are available.

Figures 1 and 2 were produced using a full epistasis mode of resistance but the results are qualitatively similar for all three epistasis modes (Figure 3). The number of drugs used does not qualitatively change the results reported in Figure 1: using more drugs will increase the useful therapeutic life proportionally for each policy. Deploying more drugs in MFT will also increase the “tipping point” where the advantage of mutations encoding drug resistance is greater than the fitness cost [41], allowing increased drug usage without significant resistance spread.

Multiplicity of infection is an important factor in determining the length of effective drug use as all policies last longer with lower MOI (Figure 1). Its impact is mediated by two factors: increasing prevalence of resistance, and the impact of recombination. The “prevalence” of resistance (the proportion of treated humans with one or more resistant clones) increases with MOI [13] allowing a higher frequency of resistant genotypes to be transmitted to the next generation though ‘competitive release’. However, this effect is potentially offset by a second factor: increased recombination and breakage of LD that will occur in high MOI settings.

The spread of resistance is faster in environments with weak epistasis modes. Figure 3 exemplifies this as both MFT and sequential application last longer with full epistasis than with mixed mode. In fact, the modes of epistasis are more important in determining policy duration than the policies themselves. In mixed mode, the main locus is sufficient to confer resistance in half of the environments treated with a drug so there is no need for association with a second locus. This is quantitatively more important if high fitness penalties are associated with resistance mutations: the second locus encoding resistance to a drug is not needed in half of the drug treatments (in contrast to full epistasis where it is always needed), therefore it is often deleterious even in the presence of a drug.

**Figure 4:**
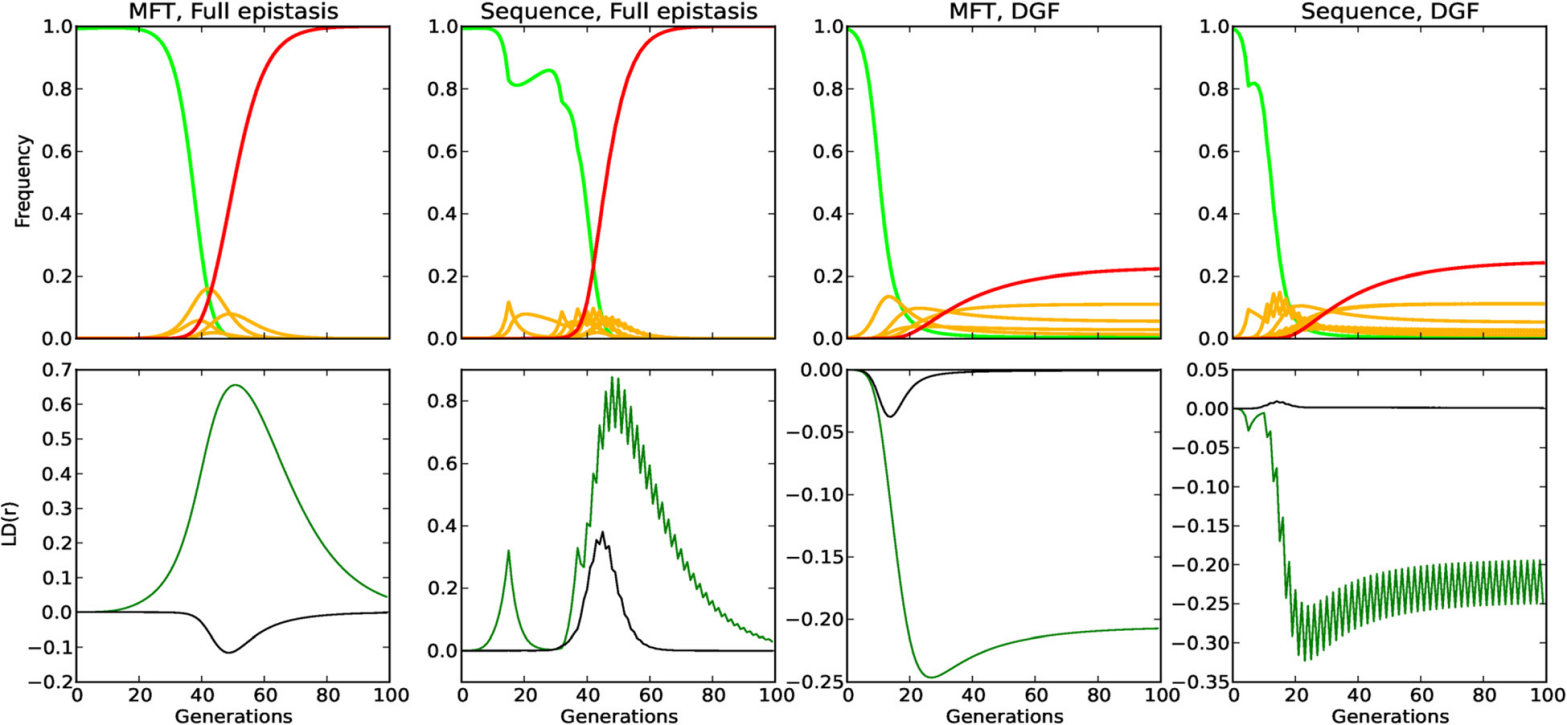
**Frequency and linkage disequilibrium,**_**(r)**_**, with varying policies and epistasis modes for a drug usage of 30% and fitness penalty of 10%.** Each column depicts a different policy (MFT or sequential application) and epistasis mode (full epistasis or DGF). The top row shows the frequency over time of all genotypes: green is the fully sensitive genotype, red is the fully resistant genotype, and yellow lines represent genotypes containing one, two or three resistance mutations. The bottom row plots LD between two loci encoding for the same drug (green) and two loci encoding resistance to different drugs (black). Note that the Y scale for LD (_r_) is different on each column.

Sexual recombination reduces any statistical association (LD) between resistant alleles at different loci. The frequency of resistance qualitatively influences the impact of recombination. One of the fundamental assumptions in any malaria model of resistance is that the frequency of resistant alleles is less than 50% (as WHO policies postulate efficacy levels above 90% [7]). If a clone is resistant to one drug, a recombination event involving a different clone will probably generate offspring that are only resistant to the same drug, as the other clone is probably sensitive (due to the assumption of low frequency of resistance). Linkage disequilibrium patterns can change with different epistasis modes and drug policies. Figure 4 shows the LD (r) for both policies assuming full epistasis or DGF over the whole 200 generations simulated. Both the signal and magnitude of LD varies with epistasis and policy: it is positive in full epistasis for loci involved in resistance to the same drug and negative for DGF. From an empirical perspective, all signals and intensities of LD are plausible depending on policy and epistasis mode [13]. Note that positive LD indicates resistance alleles are found together in the same parasite genotypes more often than expected by chance, and negative LD indicates that they are associated less often than expected.

The fundamental consequences of different drug deployment policies can therefore be summarized on Table 1 as follows. Sequential Application is characterized by medium drug therapeutic lifespans and low levels of multi-drug resistance. MFT is characterized by long drug therapeutic lifespans and low levels of multi-drug resistance. Combination therapy is characterized by the longest drug therapeutic lifespans but extreme increases in multi-drug resistance.

**Table 1:** Summary of the impact of combination therapy, MFT and sequential application on useful therapeutic life and the spread of multi-resistant genotypes assuming drug usage typical in control scenarios

***Policy***	***Impact***
Sequential application	Medium useful therapeutic life
	Low multi-drug resistance
MFT	Long useful therapeutic life
	Low multi-drug resistance
Combination therapy	Longest useful therapeutic life
	Extreme increase in multi-drug resistance

## Discussion

The key conclusion is that MFT policies last much longer than sequential application in areas with low to medium levels of drug usage; sequential deployment is marginally better once treatment rates become substantial (>~45% in the simulations; Figures 1 and 3). MFT is therefore likely to be particularly effective in areas of moderate to intense transmission because such areas typically contain a relatively large proportion of semi-immune individuals able to harbour asymptomatic infections (repeated infection leads to the development of clinical immunity [42,43]) meaning that drug treatment rates will tend to be lower and recombination rates higher.

MFT and sequential application show similar patterns of spread for multi-drug resistant genotypes up to the 10% levels of resistance regarded as maximum permissible in WHO policies (with MFT being slightly better). This is in contrast to the end-of-life profile of combination therapies. However, if the frequency of resistance increases substantially above WHO standards then the MFT multi-resistance profile shifts considerably (Figure 2B). Therefore a realistic assessment of the ability to maintain resistance within WHO policy limits should be a fundamental decision guideline regarding the introduction of MFT.

The results are far less favourable to MFT than the previous analyses by Boni *et al.*[9]. Their analyses embedded genetics into an overall model of malaria epidemiology so had to simplify the analysis of the former. In particular their treatment of recombination assumed random mating among parasites whereas it can only actually occur between clones co-infecting the same human, and they also ignored the potential impact of ‘competitive release’ (see Background). In addition, their parameter space assumed many factors were independent whereas they are actually correlated. Increasing R0, for example, will increase MOI and hence both the amount to recombination and level of competitive release; increasing the fraction of infections treated will decrease the amount of recombination, and so on. The present study takes the simpler approach of isolating the genetic component and ignoring the complexities of the underlying malaria epidemiology; this has the advantage of transparency and also avoids the complexity of having to calibrate the epidemiology, including such factors as the acquisition of immunity. The key genetic factor in our simulations is MOI, a factor easily measured in the field, as an external input into the simulations; critically, MOI determines levels of competitive release and recombination, the latter being accurately calculated as only able to occur between clones taken up by a mosquito from the same human, while drug treatment rate and factors such as natural selection against mutations are also externally defined. The analysis are probably not too dissimilar, Boni *et al.* noting in their Appendix that “MFT seems to enjoy the broadest advantage when the cost of resistance is high and the fraction of cases treated is low”; the first factor, natural selection, is intuitive (as for example on Figure one of [33]) while the second is consistent with the results presented here in Figures 1 and 3. The main difference is that MFT was found to be slightly counter-productive at high levels of drug treatment, a result that can most likely be attributed to the inclusion of ‘competitive release’ and recombination being restricted to clones taken up by the same mosquito. It is therefore important to note that current control and elimination agendas are explicitly aimed at identifying and treating all malaria infections (i.e., severely increasing drug usage) [44] and that the results regarding useful therapeutic life are partially reversed with high drug usage (Figures 1 and 3) although the difference between policies at higher drug usage is relatively smaller than the difference at low to medium drug usage.

This strategy of isolating the genetic processes from malaria epidemiology has the advantages of clearly focussing on the genetic of resistance (the spread of resistance is, by definition, a genetic process) while not precluding it being set within an epidemiological context: it simply forces the user to be explicit about how the epidemiological factors affect the genetic parameters. As a concrete example, deploying drugs might plausibly decrease transmission intensity and therefore might decrease MOI. This putative reduction in transmission may also decrease immunity levels which might, depending on local diagnostics, increase the levels of drug use as symptomatic infections become more common. The key point is that all the epidemiological factors need to be carefully calibrated, validated and converted into genetic parameters before being brought into a larger genetic/epidemiology model. Note also that it is not simply their initial values that need to specified, but their dynamic change in response to drug deployment and spread of resistance that needs to be specified. It is highly debatable whether there is sufficient clinical or field data to calibrate these processes in a compelling manner, hence the focus here is on the genetics and the use of summary epidemiological measures such as MOI.

A recent study [45] took almost the opposite approach: it included immunity but ignored most of the interesting genetics, superinfection, intrahost competition and, critically, the effects of recombination in the sexual phase. It mainly focused on time until resistance emerges in a population and revisited previous work [46] that used essentially the same technique i.e. survival probabilities calculated using branching processes based on negative binomial distributions. As would be expected intuitively, the probability of a novel resistant mutation surviving, and hence waiting time until it occurs, is dependent on their selective advantage (Box two and Figure two of [46]). Waiting times could therefore be easily estimated using the explicit genetic model described above to calculate selective advantages and hence the probabilities of survival; these are relatively easy to compute or could be estimated from the relationship given in Box two of [46]. Survival probabilities were not considered here because it is not certain that resistance will emerge from within the treated population (immigration may be a bigger threat [39]) but, more importantly, it is likely that some level of resistance will already be present to most of the candidate partner drugs within an MFT, so it is MFT’s impact in delaying the spread of resistance, compared to a policy of rotation, that is likely to be the key operational question.

A distinction should be made between formal policy (which is currently sequential deployment virtually everywhere) and the pragmatic realities of different countries and regions. Distribution of designated ACT through the formal health sector is the official policy in most countries but the private and informal sectors still distribute numerous types of ACT plus CQ and SP [8], therefore the *de facto* reality is likely to be MFT in many areas. The analyses and results indicate that the MFT approach adopted by the private sector is unlikely to undermine official drug policy so there would be little justification for attempting to suppress drug distribution through this sector (with the obvious caveat that distribution of artemisinin monotherapies and/or ineffective drugs should be suppressed). It should be noted, however, that intense MFT deployment in some areas, likely to be urban settings with high levels of informal drug provision, is likely to change MFT into a *de facto* policy of combination therapy (due to long drug half-lives) so there is a very real of danger of driving local epidemics of multi-drug resistance malaria in these settings (Figure 2).

Residual drug levels have little impact on sequential policies because the residual and treatment drugs are assumed to be the same. They do have an effect in MFT policies as parasites may have to survive one drug at residual levels, and survive treatment by another drug, which, as noted before, constitutes a type of combination therapy. This important effect has not been explicitly quantified for several reasons: (i) the proportion of people with residual drug treatment depends on the overall drug use which consists of treatment against malaria infection, and of presumptive treatment of people who have malaria symptoms (e.g., fever) but are not actually infected; (ii) presumptive treatment is much more common in high transmission (high MOI) areas with poor clinical diagnosis, so higher MOI settings show a positive correlation, and hence confounding, between lower therapeutic drug usage (due to immunity) but higher residual drug levels (due to high presumptive treatment); (iii) residual drug levels may require weaker epistatic models than therapeutic drug use; (iv) recent attempts to improve diagnosis using rapid diagnostic tests may greatly reduce presumptive drug use; and (v) finally, different MFT implementations can alter the effects of residual drugs: if an MFT implementation can assure that the same individual is always treated with the same drug (either by tracking each individual treatment history or, more pragmatically, by having different therapies at different geographical locations) then the impact of residual drug levels will be much reduced; note however that such schemes would have to meet the clinical requirement that a patient failing treatment would have to be retreated by a different drug. Consequently an indirect argument for the effects of residual drugs on MFT is employed: the difference between MFT and CT was small and CT was generally slightly better at maximizing therapeutic lifespan (e.g., Figure 1) so it is concluded, while noting the threat of multi-drug resistance (Figure 2), that the fact that MFT may operationally merge into a type of CT would not undermine its deployment and may even act as a kind of cost effective way of harnessing the benefits of CT without the financial and technical penalties of having to co-formulate drugs into a CT.

Compliance with treatment guidelines is fundamental to delay the spread of drug resistance. Different epistasis modes were interpreted as reflecting varying immunity profiles, but epistasis can also reflect treatment compliance [13]. Full compliance is represented by strong epistasis (full treatment forces the parasite to have all resistant alleles in order to survive) and poor compliance is represented by weaker epistasis modes. Figure 3 clearly shows that stronger epistasis (full compliance) allows both policies to last longer. The model provides strong support for the importance of ensuring proper dosing and compliance to slow the spread of drug resistance. An inherent drawback of MFT is a possible proliferation of packaging with subsequent confusion over regimens. As a plausible example, if three drugs are used in an MFT, each drug is supplied by two manufactures, and there are four dosing regimens for each drug (one adult and three paediatric), this makes 24 packages in total; the dosing regimens may also differ (two treatments per day for artemether-lumefantrine, once per day for most other ACT). There is clearly considerable scope for confusion so it is therefore strongly recommended that any deliberate policy of MFT be accompanied by dedicated education programmes to ensure adequate dosing to offset the threat of resistance.

## Conclusions

MFT out-performs the standard policy of sequential application for realistic model parameters provided treatment rates are less than about 50%, thereafter MFT performs slightly worse than other policies. The results suggest that the impact of MFT on the spread of multiple resistant genotypes is negligible or even slightly better than sequential application as long as resistance is in within WHO guidelines. An important conclusion is that widespread availability of multiple ACT through the informal sector is not an immediate cause for alarm (although artemisinin monotherapies or low quality drugs should definitely be suppressed) and that countries may reasonably choose to deliberately employ a policy of MFT within the public health sector.

## Competing interests

The authors declare that they have no competing interests.

## Authors' contributions

TA and IMH developed the model. TA implemented the model, carried out the simulations and collated the results. Both authors wrote, read and approved the final manuscript.
